# Nanometer-Scale Vibration Measurement Using an Optical Quadrature Interferometer Based on 3 × 3 Fiber-Optic Coupler

**DOI:** 10.3390/s20092665

**Published:** 2020-05-07

**Authors:** Soongho Park, Juhyung Lee, Younggue Kim, Byeong Ha Lee

**Affiliations:** School of Electrical Engineering and Computer Science, Gwangju Institute of Science and Technology, 123 Cheomdangwagi-ro, Buk-gu, Gwangju 61005, Korea; shpark88@gist.ac.kr (S.P.); kendo1009@gist.ac.kr (J.L.); younggue92@gm.gist.ac.kr (Y.K.)

**Keywords:** optical quadrature detection, optical interferometer, vibration measurement, displacement measurement, 3 × 3 fiber-optic coupler, Lissajous curve, ellipse fitting method

## Abstract

We propose a nanometer-scale displacement or vibration measurement system, using an optical quadrature interferometer and the post-processing technique that extracts the parameters necessary for characterizing the interferometric system. Using a 3 × 3 fiber-optic coupler, the entire complex interference signal could be reconstructed with two interference signals measured at two return ports of the coupler. The intrinsic phase difference between the return ports was utilized to obtain the quadratic part of the interference signal, which allowed one to reconstruct the entire complex interference signal. However, the two measured signals were appreciably affected by the unequal detector gains and non-uniform intrinsic phases of the coupler. Fortunately, we could find that the Lissajous curve plotted by the two signals of the interferometric system would form an ellipse. Therefore, by fitting the measured Lissajous curve to an ellipse, we could extract the parameters characterizing the actual system, which allowed the nanometer-scale measurement. Experimental results showed that a 20 kHz sinusoidal vibration with an amplitude of 1.5 nm could be measured with a standard deviation of 0.4 nm.

## 1. Introduction

With the development of technology, more precise and accurate techniques for measuring smaller displacement and vibration are required from scientific research fields to various industrial fields, including metrology, non-contact ultrasound and/or pressure measurement [1,2,3]. Most conventional nanometer-scale precision measurement techniques have used the optical quadrature fringe detection method implemented with various interferometers [4,5,6,7,8,9,10]. In principle, if the entire complex interference signal is available, a pair of conjugate signals, for example, the phase information of the interference signal can be easily extracted, with which the displacement applied to the interferometer is simply calculated. However, due to the nature of the typical photodetector, it can only obtain a part of the entire complex interference signal, i.e., the amplitude part [11,12] in general. Thus, a method for obtaining the remaining part of the signal, the phase part, needs to be devised. 

Theoretically, a pair of conjugate signals, the in-phase and quadrature components, can be extracted from any two signals having a mutual phase difference, but not an integer multiple of π. For this reason, many studies have attempted to obtain the interference signals of different phases, using bulk optic components such as polarization optics and electric modulators (such as acousto-optic modulators or electro-optic modulators) [8,9,10,13]. However, these methods suffer from drawbacks of electromagnetic interference and signal drift, due to the use of electric components. Usually, they require sophisticated optical alignments. The interference signals used to extract the conjugate signal pair are asked ideally to have (a) zero-offsets, (b) equal amplitude coefficients, and (c) well-defined phase difference between each other. However, in the actual system, difficulties arise from electrical DC-offsets, unequal detector gains, and drift in the phase difference. Thus, there are errors of several nanometers in displacement measurements [4,5,14,15].

In the 3 × 3 fiber-optic coupler-based interferometer, the interference signals, simultaneously measured at the three return ports, have unique intrinsic phases, depending on the structural characteristics of the fiber coupler [2,11]. An ideal 3 × 3 coupler with equal power splitting ratios has the same intrinsic phase difference of 2π/3. However, it is difficult to fabricate the ideal coupler, due to errors in the manufacturing process. For the coupler with different splitting ratios, the intrinsic phase difference caused them to not be the same as each other, but the phase difference among the return ports can be theoretically calculated with the experimentally measured splitting ratios of the coupler [11]. However, the precision of measuring the splitting ratio of the coupler cannot be guaranteed to be sufficiently high enough to get a precise quadrature detection. In this study, we present a 3 × 3 fiber-optic coupler-based interferometer system that is capable of doing accurate optical quadrature detection, despite not meeting the preconditions (a)–(c).

As a main point of this study, we present that the relationship between the two interference signals, measured at any two return ports of the interferometer system not meeting the preconditions (a)–(c), can be expressed with an equation of ellipse. Therefore, by fitting a curve to the signal of an actual non-ideal system, we can retrieve the information of the system, deviated from the ideal one meeting the preconditions (a)–(c), with which we can continue the quadrature detection process. To the best of our knowledge, this is the first application of the curve fitting approach in the displacement measurement system, based on a 3 × 3 coupler-based interferometer. To verify the performance of the proposed method, we measure the sinusoidal nanometer-scale oscillation driven by a piezoelectric transducer (PZT) and present the performance of measurement. The stability of the system parameters is investigated by measuring the variations in the amplitude coefficients of the two interference signals and their intrinsic phase difference for three hours at every 30 s. 

## 2. Principle of Optical Quadrature Measurement and Ellipse Fitting

In the fiber-optic coupler-based interferometer, the phase of the interference signal at the return port depends on the position of the port and the characteristics of the coupler, especially the total number of ports and the power splitting ratios. The 3 × 3 fiber coupler with equal power splitting ratio has an ideal intrinsic phase difference of 2π/3, which makes it possible to extract the quadrature component of each interference signal. However, the intrinsic phase difference depends on the actual power splitting ratios of the coupler. It is noteworthy that in a 2 × 2 coupler, the phase difference between two return ports is always π, regardless of its power splitting ratio. 

**Figure 1:**
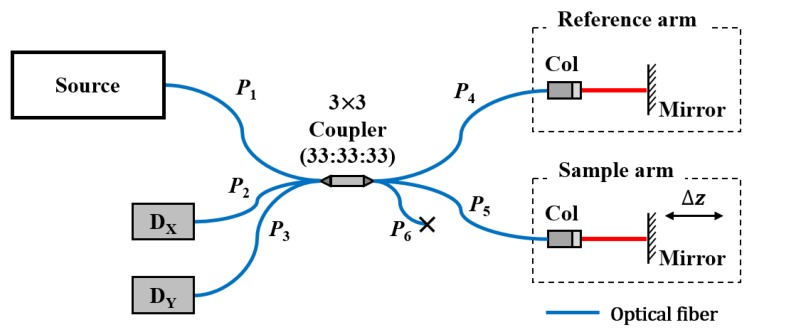
Schematic of the Michelson interferometer, based on a 3 × 3 fiber-optic coupler. Col: collimator, D: photodetector, P: fiber-optic coupler port.

Figure 1 shows a Michelson interferometer based on a 3 × 3 coupler. The input light is delivered to the reference and sample arms through the coupler, and then reflected and back-coupled into the coupler. Thereafter, the back-coupled light is measured at the return ports *P_2_* and *P_3_*, with detectors D_X_ and D_Y_, respectively. When the optical path-length difference (OPD) between the reference and sample arms is shorter than the coherence length of the light source, both detectors give the interference signals of:

(1) $$I_{X}=h+a\cos\phi ,$$

(2) $$I_{Y}=k+b\cos\left(\phi +\delta \right).$$

where, h and k are DC-offsets, and a and b are the AC amplitudes (called the amplitude coefficients) of the detected signals. The intrinsic phase difference between two return ports is denoted by δ. The phase ϕ is the result of the interference between the sample and reference arms of the interferometer, which is given by the OPD (Δz), the wavenumber ($k_{0}=2\pi /\lambda_{0}$) of the light, and the initial phase ($\phi_{0}$) of the interferometer setup as:

(3) $$\phi =2k_{0}\Delta z+\phi_{0},$$

where the factor of 2 is due to the roundtrip. At port *P_3_*, due to the intrinsic property of the coupler, the phase of the interference signal is shifted by δ, as in Equation (2).

By taking squares of $I_{X}$ and $I_{Y}$ in Equations (1) and (2), and then adding them together, we can show that the two measured signals form an ellipse equation, as given by [16]:

(4) $$\frac{{\left(I_{X}-h\right)}^{2}}{a^{2}}+\frac{{\left(I_{Y}-k\right)}^{2}}{b^{2}}-\frac{2\left(I_{X}-h\right)\left(I_{Y}-k\right)\cos\delta }{ab}=\sin^{2}\left(\delta \right).$$

The equation depends on the five parameters: the DC-offsets (*h* and *k*), AC amplitudes (*a* and *b*), and intrinsic phase difference (δ). Therefore, by changing ϕ more than 2π, with shifting the Δz of Equation (3), we can experimentally obtain the Lissajous curve of the two correlated signals, $I_{X}$ and $I_{Y}$. From the Lissajous curve, by fitting with an ellipse, the five parameters of the interferometer system can be derived. Of course, with these parameters, we want to get the Δz with a higher precision and an initial phase-independent uniform sensitivity.

For the curve fitting, at first, the data is fitted using a polynomial equation. By expanding the terms of Equation (4) and redefining the two variables as $x\equiv I_{X}$ and $y\equiv I_{Y}$, we have the general ellipse equation, in the form of a second order polynomial, as [5]:

(5) $$x^{2}+\mathrm{Bxy}+Cy^{2}+\mathrm{Dx}+\mathrm{Ey}+F=0,(B^{2}-4C<0).$$

It is not difficult to show that the five coefficients (*B*, *C*, *D*, *E,* and *F*) of the equation are mathematically related to the five parameters of Equation (4) as:

(6) $$h=\left(2\mathrm{CD}-\mathrm{BE}\right)/\left(B^{2}-4C\right),$$

(7) $$k=\left(2E-\mathrm{BD}\right)/\left(B^{2}-4C\right),$$

(8) $$a={\left[\frac{\left(h^{2}+\mathrm{Bhk}+Ck^{2}-F\right)}{1-B^{2}/\left(4C\right)}\right]}^{1/2},$$

(9) $$b=a/\sqrt{C},$$

(10) $$\delta =\cos^{-1}\left(\frac{-B}{2\sqrt{C}}\right).$$

**Figure 2:**
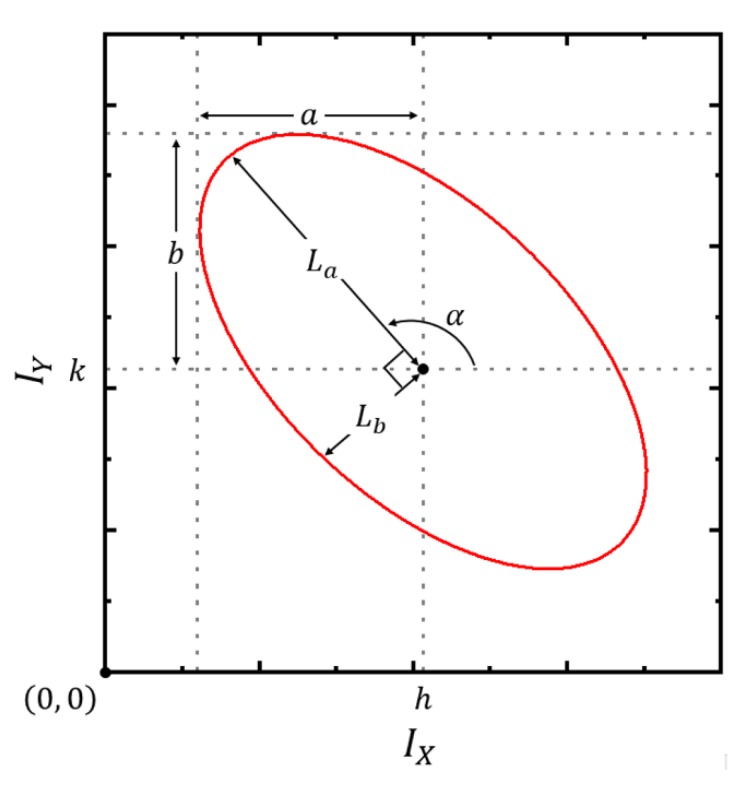
Lissajous trajectory of the data sets measured at two photodetectors, when the optical path-length difference (OPD) changes by 2π in Figure 1. a and b are the AC amplitudes of the interference signals, and $L_{a}$ and $L_{b}$ are the semi-major axis and semi-minor axis of the ellipse; α is the rotating angle.

For better visibility of the ellipse curve fitting, the ellipse can be expressed using conventional parameters; semi-major axis ($L_{a}$), semi-minor axis ($L_{b}$), and rotation angle (α), as in Figure 2.

By defining the two intermediate parameters as:

(11) $$\begin{array}{l} G=\frac{2\left(\mathrm{BDE}+4\mathrm{CF}-E^{2}-CD^{2}-FB^{2}\right)}{B^{2}-4C},\\ H=\sqrt{B^{2}+{\left(1-C\right)}^{2}}\;, \end{array}$$

the parameters of the ellipse in Figure 2 can be obtained as [17]:

(12) $$L_{a}=\sqrt{\frac{G}{1+C-H}},$$

(13) $$L_{b}=\sqrt{\frac{G}{1+C+H}},$$

(14) $$\alpha =\frac{1}{2}\left[\pi +\cot^{-1}\left(\frac{1-C}{B}\right)\right].$$

It is noted that the DC-offsets are the same as Equations (6) and (7). In addition, the ellipse eccentricity (ε) is calculated as:

(15) $$\varepsilon =\sqrt{1-{\left(\frac{L_{b}}{L_{a}}\right)}^{2}}=\sqrt{\frac{2H}{1+C+H}}.$$

With the parameters of Equations (6)–(10), derived from the curve fitting, the phase ϕ of the interference signal can be calculated. By using the cosine subtraction formula with Equations (1) and (2), we obtain [16]:

(16) $$\sin\phi =\frac{b\left(I_{X}-h\right)\cos\delta -a\left(I_{Y}-k\right)}{\mathrm{ab}\sin\delta }.$$

Alternatively, for the continuous phase information, by dividing it with Equation (1), we have a tangent function of the phase:

(17) $$\tan\phi =\frac{b\left(I_{X}-h\right)\cos\delta -a\left(I_{Y}-k\right)}{b\left(I_{X}-h\right)\sin\delta }.$$

Therefore, the small change in the sample arm of the interferometry system, the displacement giving d(Δz), can be calculated with the phase variation dϕ of Equation (17), by using Equation (3) as:

(18) $$d\left(\Delta z\right)=\frac{1}{2k_{0}}d\phi =\frac{\lambda_{0}}{4\pi }d\phi .$$

From another point of view, if we have only a single channel for detecting the interference signal such as the one of Equation (1), for the same displacement d(Δz), the detected signal intensity $dI_{X}$ becomes dependent, not only on the amount of phase variation dϕ, but on the initial phase including $\phi_{0}$, at which the small displacement begins. Thus, to get the initial phase independent uniform sensitivity, we need at least one more signal channel having a different initial phase such as Equation (2). For that case, however, the relative phase δ and the AC signal amplitudes, *a* and *b*, of Equations (1) and (2) should be adjusted with hardware to have preset ideal values, or they should be precisely extracted with a premeasurement made with the same hardware. This premeasurement can be done effectively by utilizing the proposed ellipse curve fitting method.

## 3. Experimental Methods

### 3.1. Parameter Extraction with Ellipse Fitting Method

**Figure 3:**
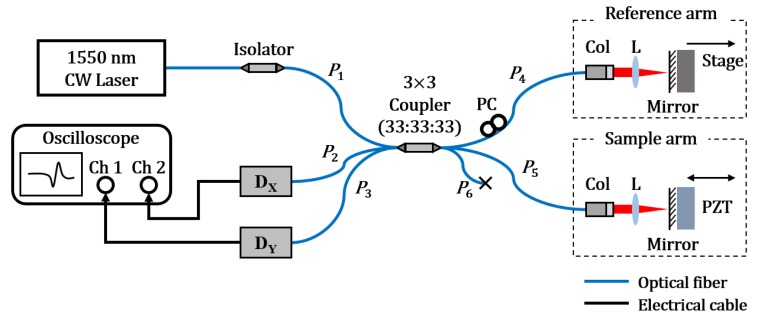
Displacement measurement system implemented with a 3 × 3 fiber-optic coupler-based interferometer. Col: collimator, D: photodetector, L: lens, P: fiber-optic coupler port, PC: polarization controller, PZT: piezoelectric transducer.

The Michelson interferometer based on a 3 × 3 fiber-optic coupler (R315009004153, Flyin Optronics Co., Ltd., Shenzhen, China) was constructed as shown in Figure 3. A polarized and stabilized 1550 nm laser (SFL1550P, Thorlabs. Inc., Newton, NJ, USA) was used as the light source. The visibility of the interference signal was maximized by adjusting the polarization controller in the reference arm, which was thought for the beams in both arms to give the same polarization states.

In order to check the relationship between the interference signals, measured by the two photodetectors (PDB460C, Thorlabs. Inc., Newton, NJ, USA), the mirror in the reference arm was moved using a linear stage by more than λ/2 along the optical axis. The interference signals were acquired by an oscilloscope (DSO6054A, Agilent Technologies. Inc., Santa Clara, CA, USA), with which the Lissajous curve, similar to Figure 2, was plotted. It is noted that one revolution (2π phase change) from any reference point on the curve is equal to the λ/2 OPD change in Equation (18). In order to find the parameters of Equations (1) and (2) from the measured data, the ellipse fitting of Equation (5) was performed.

### 3.2. System Stability Measurements

The stability of the proposed interferometer system was verified by checking the time variations of the fitting parameters extracted in Section 3.1. The same measurements were performed with a 30 s interval for 3 h (360 measurements in total). The time variations of the five fitting coefficients of Equation (5) were calculated, and the other five ellipse parameters of Equations (6)–(10) were calculated and analyzed.

### 3.3. Small Displacement Measurement

To verify the proposed optical quadrature detection method for a small displacement or vibration measurement, the OPD of the interferometer was slightly changed, by oscillating the mirror attached to the PZT in the sample arm. A small sinusoidal displacement (~ nm) was applied to the mirror by deriving the PZT with a voltage of 500 mV*_pp_* amplitude and 20 kHz frequency, using a function generator.

## 4. Results

### 4.1. Parameter Extraction and Conjugate Signal Pair Reconstruction

**Figure 4:**
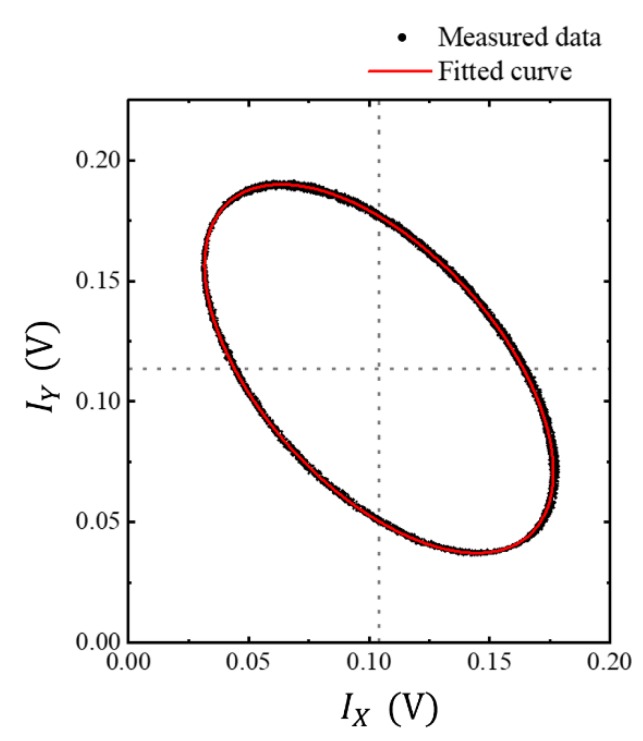
Lissajous trajectories of the data sets measured simultaneously at two photodetectors, with the OPD change in a 3 × 3 fiber-optic coupler-based interferometer. The trajectories of the data sets correspond to 10 revolutions. The measured data points are indicated by black dots and the red solid line is the fitted ellipse curve.

The OPD of the interferometry system was linearly changed to give approximately 10 revolutions along the Lissajous curve. As shown in Figure 4, the data points of the 10 revolutions were well located along a curve, which was well fitted with an ellipse.

The curve fitting with the ellipse was carried out using the OriginPro (OriginLab. Inc., Northampton, MA, USA), which was a program based on an orthogonal distance regression algorithm. With the obtained fitting coefficients, *B* to *F*, of Equation (5), the measurement parameters of Equations (1)–(4) were calculated and listed in Table 1.

**Figure 5:**
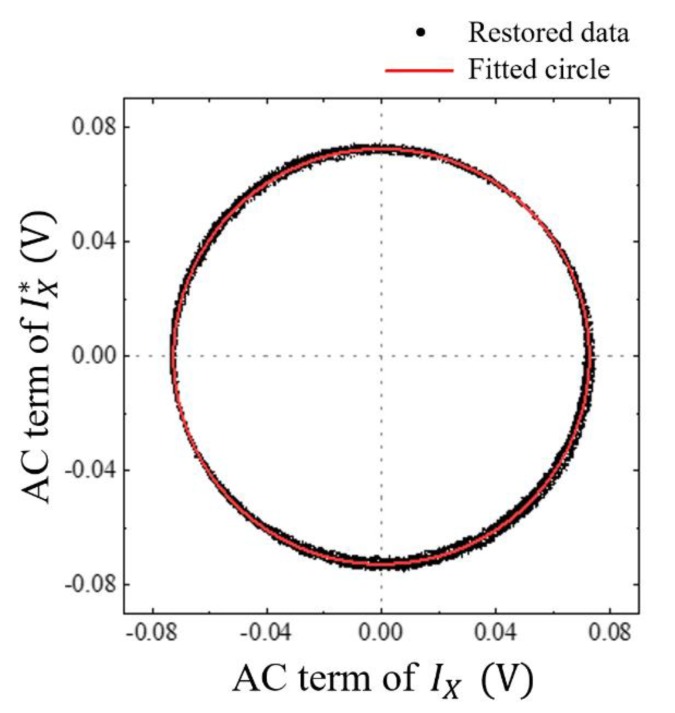
Lissajous curve replotted with the reconstructed conjugate signal pair. The black dots are the reconstructed data sets and the red solid line is the fitted curve.

Furthermore, to check the performance of the proposed method, the quadrature signal ($I_{X}^{*}$), the sine version of the original cosine signal ($I_{X})$ in Equation (1), was constructed by using the parameters of Table 1 and the phase ϕ calculated with Equations (16) or (17). With the reconstructed conjugate signal pair, the Lissajous curve was replotted. As shown in Figure 5, it was fitted well with a circle, giving a *R^2^* value of 0.9999, which confirmed that the entire complex interference signal was restored successfully with the proposed curve fitting method.

### 4.2. Stability

The two interference signal data were collected every 30 s, then the Lissajous curve in Figure 4 was plotted. The fitting parameters of Equation (5) were calculated for three hours and listed in Table 2.

With these curve fitting parameters, the measurement parameters of Equations (1) and (2), or (4), were also calculated by using Equations (6)–(10) and listed in Table 3.

We can see that there were no significant differences between Table 1 and Table 3, which means that there was no significant change in the stability of the system during the 3-h time period. The biggest change was in the AC signal amplitude. Particularly in the AC amplitude coefficient *a* of the first detector (D_X_), the standard deviation (STDEV) was increased by about 50% with the 3 h period. However, there was no appreciable change or drift with time in the intrinsic phase difference δ.

The STDEV of δ was 0.0049, indicating that the phase drift over 3 h was less than 0.28°. The drift in δ was calculated to correspond to the displacement deviation of 0.6 nm. The measurements were performed in a laboratory environment at room temperature. In the experiment conducted by Choma et al. [11], the phase error was approximately 2.5%. Compared to their study, our measurements were 10 times more stable and accurate. They believed that the intrinsic phase error was attributed mainly to the variation in the splitting ratio of the coupler with temperature [11]. However, variations in the electrical equipment (such as unequal gains of detectors and their drift) must be considered as well.

We also calculated the ellipse parameters of Figure 2 and the two intermediate parameters (*G, H*) to confirm the stability and characteristics of the fitted ellipse. As presented in Table 4, the fitted ellipse was inclined by 132.38° and had a $L_{a}$ of 0.0940 and $L_{b}$ of 0.0497. The evident change was in $L_{b}$. Its STDEV for three hours of measurements was 0.9%. However, the angle of the ellipse had the smallest variation, only 0.2% over three hours, corresponding to 0.26°.

### 4.3. Small Displacement Measurement

**Figure 6:**
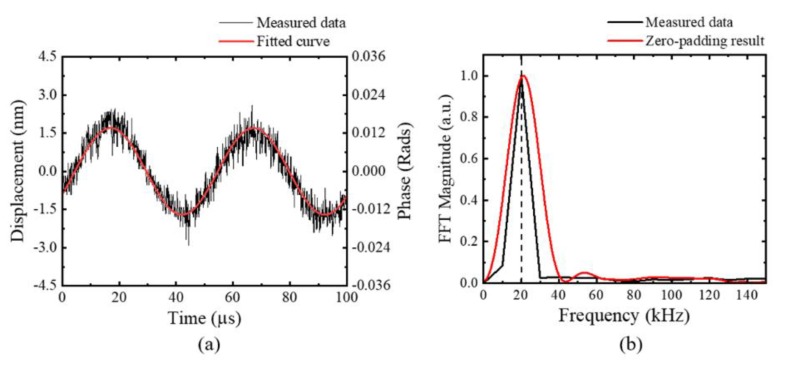
Small displacement measurement; (**a**) the extracted displacement signal (black dots) and the fitted sinusoidal curve (red solid line), and (**b**) its FFT signal (black) and zero-padding signal (red). A 20 kHz sinusoidal oscillation was applied to the sample arm with a PZT.

After obtaining the parameters of the system with the premeasurement and the proposed ellipse curve fitting method as shown in Table 1, the main small displacement measurement was performed. Figure 3 shows the experimental setup, wherein a 20 kHz sinusoidal motion was induced in the PZT of the sample arm using a function generator, while the reference arm was fixed. A small alternating voltage of 500 mV*_pp_* was applied to the PZT. Thereafter, the entire complex interference signal was reconstructed using the fitting parameters listed in Table 1, and the PZT-induced displacement was calculated by using Equations (17) and (18). As shown in Figure 6a, the amplitude of the measured phase variation was about 0.012 radians, corresponding to 0.69° in the phase angle, from which the maximum displacement of the sinusoidal vibration was calculated to be around 1.5 nm. The STDEV between the measured data and the fitted sinusoidal red curve was 0.4 nm. In this experiment, any filter for decreasing the noise was not applied. In addition, a frequency analysis was performed by taking the FFT to confirm whether the displacement signal was identical to the motion induced by the function generator. Figure 6b shows the frequency spectrum of the measured displacement, which had a dominant peak at 20 kHz. However, when the zero-padding was performed to monitor the frequency component more smoothly, the peak frequency was slightly blue-shifted. We infer that this shift was probably due to the zero-padding and the associated signal filtering process. Further analysis is necessary.

## 5. Discussion

There are other methods for calculating the intrinsic phase difference, such as that of Choma et al. [11], who experimentally measured the power splitting ratio of the coupler. However, since the intensity of the interference signal is easily affected by the experimental environment, the proposed curve fitting method would be more accurate and convenient in obtaining the parameters of the interferometer system. Moreover, it can also be used as a means for time calibration. Just sweeping the OPD of the interferometer by more than λ/2 the calibration can be completed.

The measurements in Figure 4 were made with the linear displacement corresponding to 10 revolutions in the Lissajous curve. The same measurement can be made with a small linear displacement corresponding to just one revolution. Experimentally, we observed that the STDEVs in Table 1 became smaller with increasing the number of revolutions. However, with the 3-hour experiment, the STDEVs became bigger with time, as can be seen in Table 1 and Table 3.

The evident factor that caused the error in the displacement measurements was the high frequency component of the signal. However, in our experiments, no filter was used to cut the high frequency component. Depending on the application, a proper analog or digital filter, such as a low or band pass including the notch filter, is expected to decrease the noise of the measurement significantly.

Due to the high electric noise, inducing a tiny but highly calibrated linear displacement was not easy. Therefore, the minimum noticeable sinusoidal vibration was applied to the sample arm of the system with PZT, and obtained the displacement amplitude of it as 1.5 nm, as in Figure 6a. Further, the STDEV between the measured data and the fitted sinusoidal curve was calculated as 0.4 nm, which is thought to be considered as the short-term sensitivity of the system. For the long-term sensitivity, the contribution of the intrinsic phase difference was more than 0.6 nm, but still in the sub-nanometer range. The signal-to-noise ratio calculated with the RMS value of the small signal giving Figure 6a and the maximum output voltage of the detector, was about 75 dB.

Further, we measured the displacement only at one point of an object. For the double points measurement, the blocked port, *P_6_* in Figure 1, could be used, which could be a subject of future work. By utilizing a ribbon fiber, a system suitable for multi-points measurements can also be implemented. The proposed technique can be used for fields requiring high precision vibration or displacement measurements. We are also seeking ways to improve this technique, so as to measure the roughness of the surface of various objects, including paintings and potteries.

## 6. Conclusions

We have demonstrated a nanometer-scale displacement or vibration measurement system implemented by using a 3 × 3 fiber-optic coupler-based interferometer. By utilizing the intrinsic phase difference of the multi-port interferometer, we could reconstruct the entire complex interference signal. Moreover, we showed that the relationship between the interference signals measured at any two return ports of the coupler could be expressed with an equation of ellipse. Therefore, the ellipse curve fitting could be applied to the Lissajous curve plotted with the two measured interference signals. The five parameters of the ellipse representing the interference system could be successfully extracted from the curve fitting. With the pre-obtained system parameters, the displacement applied on the system could be measured with a higher precision and a uniform sensitivity, furthermore, not being appreciably affected by the initial phase of the system.

The stability of the interferometer system was affected, not only by the characteristics of the coupler, but also by the experimental conditions, including the different gains and DC-offsets of the detectors. The Lissajous curve was plotted every 30 s for 3 h, by changing the OPD in the sample arm. No significant changes in the ellipse parameters were observed. The evident change happened in the length of the semi-minor axis of the ellipse $\left(L_{b}\right)$, with a standard deviation (STDEV) of 0.9% for over 3 h. However, the intrinsic phase difference of the coupler was rather stable; it had a mean of 2.1671 radians and STDEV of 0.23%, corresponding to 0.6 nm error in the displacement measurement. The orientation angle of the ellipse was also stable, only 0.26° in STDEV.

To verify the accuracy of the measurement with the proposed method, a 20 kHz small sinusoidal vibration was applied to the sample arm, with a PZT driven by a 500 mV*_pp_* voltage. The 20 kHz vibration of 1.5 nm amplitude was measured with a STDEV of 0.4 nm. The signal-to-noise ratio was about 75 dB, which is expected to be increased by using proper frequency filters depending on applications. With these results, we expect that the proposed system and the signal processing method can be easily used in many applications where precise vibration or displacement measurements are required.

## Figures and Tables

**Table 1:** Mean and standard deviation (STDEV) of the two interference signal parameters, calculated with a data set forming a Lissajous curve of 10 revolutions.

	*h* (V)	*k* (V)	*a* (V)	*b* (V)	δ (rad)
Mean	0.1041	0.1136	0.0726	0.0765	2.1685
STDEV (σ)	0.0005 (0.48%)	0.0005 (0.41%)	0.0003 (0.38%)	0.0004 (0.49%)	0.0048 (0.22%)

**Table 2:** Mean and standard deviation (STDEV) of the ellipse equation fitting coefficients *B* to *F*, collected over 3 h at every 30 s.

	*B*	*C*	*D*	*E*	*F*
Mean	1.0668	0.9023	−0.3271	−0.3139	0.0309
STDEV (σ)	0.0096 (0.90%)	0.0093 (1.03%)	0.0019 (0.58%)	0.0028 (0.89%)	0.0004 (1.20%)

**Table 3:** Mean and standard deviation of the two interference signal parameters collected over three hours.

	*h* (V)	*k* (V)	*a* (V)	*b* (V)	δ (rad)
Mean	0.1034	0.1128	0.0732	0.0771	2.1671
STDEV (σ)	0.0005 (0.47%)	0.0006 (0.50%)	0.0005 (0.62%)	0.0005 (0.66%)	0.0049 (0.23%)

**Table 4:** Mean and standard deviation of the fitted ellipse parameters, collected over 3 h.

	*G*	*H*	$L_{a}$	$L_{b}$	α (°)	ε
Mean	0.0073	1.0714	0.0940	0.0497	132.38	0.8489
STDEV (σ)	0.0001 (1.65%)	0.0090 (0.84%)	0.0005 (0.55%)	0.0004 (0.90%)	0.2622 (0.20%)	0.0020 (0.23%)
